# The *Salmonella* Effector Protein SopA Modulates Innate Immune Responses by Targeting TRIM E3 Ligase Family Members

**DOI:** 10.1371/journal.ppat.1005552

**Published:** 2016-04-08

**Authors:** Jana Kamanova, Hui Sun, Maria Lara-Tejero, Jorge E. Galán

**Affiliations:** Department of Microbial Pathogenesis, Yale University School of Medicine, New Haven, Connecticut, United States of America; University of California Davis School of Medicine, UNITED STATES

## Abstract

*Salmonella* Typhimurium stimulates inflammatory responses in the intestinal epithelium, which are essential for its ability to replicate within the intestinal tract. Stimulation of these responses is strictly dependent on the activity of a type III secretion system encoded within its pathogenicity island 1, which through the delivery of effector proteins, triggers signaling pathways leading to inflammation. One of these effectors is SopA, a HECT-type E3 ligase, which is required for the efficient stimulation of inflammation in an animal model of *Salmonella* Typhimurium infection. We show here that SopA contributes to the stimulation of innate immune responses by targeting two host E3 ubiquitin ligases, TRIM56 and TRIM65. We also found that TRIM65 interacts with the innate immune receptor MDA5 enhancing its ability to stimulate interferon-β signaling. Therefore, by targeting TRIM56 and TRIM65, SopA can stimulate signaling through two innate immune receptors, RIG-I and MDA5. These findings describe a *Salmonella* mechanism to modulate inflammatory responses by directly targeting innate immune signaling mechanisms.

## Author Summary

*Salmonella* Typhimurium, one of the main causes of food-borne illnesses, stimulates inflammatory responses in the intestinal epithelium. These responses are very important for the pathogen’s ability to secure nutrients within the intestinal tract. The ability of this pathogen to stimulate intestinal inflammation depends on a protein-delivery machine known as the type III secretion system. This system “injects” bacterial effector protein into host cells to modulate a variety of cellular functions for the pathogen’s benefit. We show here that one of these effector proteins, SopA, stimulates signaling pathways that can lead to inflammation. We report that SopA exerts its function by targeting two host E3 ubiquitin ligases, TRIM56 and TRIM65, which have the ability to enhance interferon-β expression through the innate immune receptors RIG-I and MDA5. These findings describe a *Salmonella* mechanism to stimulate inflammation by directly targeting innate immune signaling mechanisms.

## Introduction

The inflammatory response to bacterial pathogens is often the result of the stimulation of pattern recognition receptors by highly conserved bacterial products collectively known as “pathogen-associated molecular patterns” or PAMPS (e.g. flagellin, nucleic acids, or bacterial envelope components such as peptidoglycan and lipopolysaccharide) [1, 2]. These products are usually recognized by transmembrane Toll-like receptors (TLRs), cytoplasmic nucleotide oligomerization domain-like (NLRs), or RIG-I-like (RLR) receptors, leading to conserved signaling cascades that culminate in the production of pro-inflammatory cytokines [3–6]. These receptors are broadly expressed in cells of the innate immune system, which are therefore able to respond to virtually any pathogen. In the intestinal epithelium, however, signaling through these receptors is highly regulated since the intestine is exposed to high concentrations of stimulatory molecules derived from the resident microbiota, which could lead to inflammatory pathologies [6–11].

*Salmonella enterica* serovar Typhimurium (*S*. Typhimurium), a major cause of food-borne illnesses around the world, induces inflammation within the intestinal tract. Although inflammation is most often viewed as a host defense response to combat pathogen infections, for *S*. Typhimurium the host inflammatory response is required for its replication because essential nutrients and respiration substrates only become available in inflamed intestinal tissues [12, 13]. Consequently, *S*. Typhimurium has evolved specific adaptations to trigger inflammatory responses in the intestinal tract that do not depend on the stimulation of pattern recognition receptors by conserved bacterial products. Indeed, stimulation of intestinal inflammation is strictly dependent on the function of a type III secretion system (T3SS) encoded within its pathogenicity island 1 (SPI-1) [14–16]. Through the delivery of effector proteins (i.e. the exchange factors SopE and SopE2 and the phosphoinositide phosphatase SopB) with the capacity to activate Rho-family GTPases, this system stimulates signal transduction pathways that lead to pro-inflammatory cytokine production [14–18]. Once initiated by its specific adaptations, the inflammatory response to *Salmonella* is likely amplified by the stimulation of canonical innate immune receptors by conserved bacterial products (PAMPS) [19–21]. The *S*. Typhimurium type III secretion effector protein SopA is a HECT-type ubiquitin ligase, which has been shown to be required for the stimulation of inflammation in cow model of *Salmonella* infection [22–25]. However, the mechanisms by which it exerts this activity are not known. Here we show that SopA exerts its function by targeting members of the tripartite-motif (TRIM) family of host ubiquitin E3 ligases.

## Results

### SopA Interacts with TRIM56 and TRIM65

**Fig 1:**
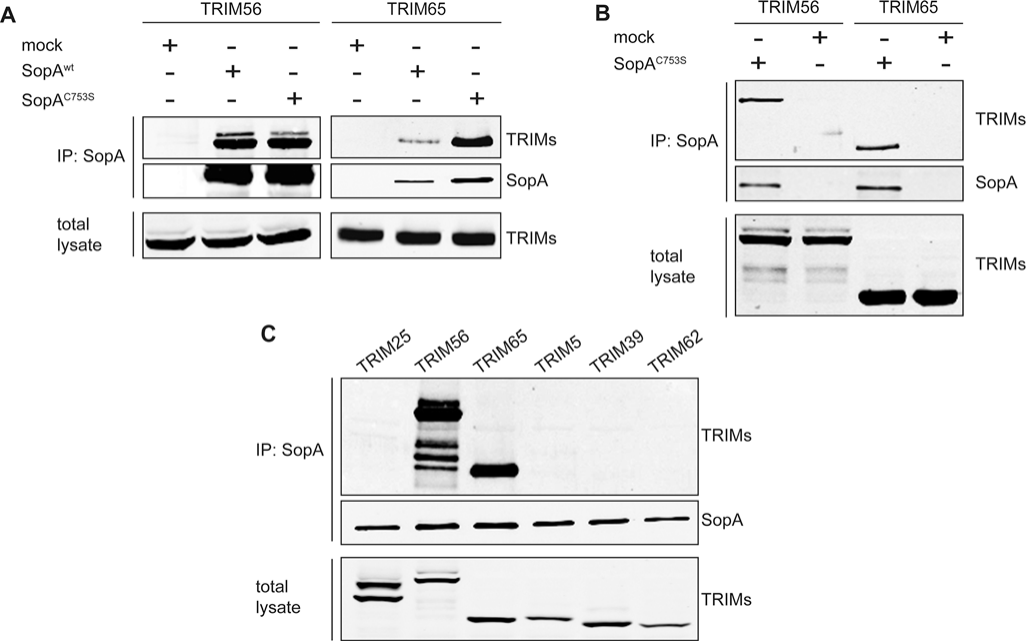
SopA interacts with TRIM56 and TRIM65. (**A**) FLAG-epitope-tagged SopA or its catalytic mutant SopA^C753S^ were transiently co-expressed in HEK293T cells with M45-epitope-tagged TRIM56 or TRIM65 and their interaction probed by immunoprecipitation and Western immunoblotting. This experiment was repeated 3 independent times with similar results. (**B**) HEK293T cells were transiently transfected with plasmids expressing M45-epitope tagged TRIM56 or TRIM65 and 18 h after transfection, cells were infected with a *S*. Typhimurium strain expressing FLAG-epitope tagged SopA^C753S^ or the equally tagged T3SS-effector protein PipA as a specificity control. Five hours after infection, cell lysates were analyzed by immunoprecipitation with anti-FLAG and Western immunoblotting with anti-M45 monoclonal antibodies. This experiments was repeated 2 independent times with similar results. (**C**) FLAG-epitope-tagged SopA^C753S^ was transiently co-expressed in HEK293T cells with M45-epitope-tagged TRIM25, TRIM56, TRIM65, TRIM5, TRIM39 or TRIM62. Cell lysates were then analyzed by immunoprecipitation with anti-FLAG and western immunoblotting with anti-M45 antibodies. This experiment was repeated 3 independent times with similar results.

In an effort to understand the mechanisms by which SopA enhances the inflammatory response to *Salmonella*, we sought to identify potential host interacting proteins. We carried out tandem affinity purification of SopA-interacting proteins from cultured human cell lysates and identified them by mass spectrometry (LC-MS/MS) analysis (see Materials and Methods). Among the proteins that specifically interact with SopA (and not with the equally processed unrelated effector protein PipA) we found UbcH7, an E2 ubiquitin-conjugating enzyme that has been previously shown to work in concert with SopA in *in vitro* ubiquitination reactions [25, 26] (S1 Fig). More significantly, this analysis also identified the tripartite motif-containing protein (TRIM) 65 (S1 Fig and also see below). We also investigated potential SopA-interacting proteins after its delivery by the *S*. Typhimurium SPI-1 T3SS in the context of bacterial infection. We infected cultured human epithelial cells with an *S*. Typhimurium strain expressing either FLAG-epitope-tagged SopA or the equally tagged unrelated T3SS effector protein PipA as a specificity control. Interacting proteins were then identified by immune-affinity purification and mass spectrometry analysis. Consistent with the tandem affinity purification results, this analysis also identified TRIM65 as a specific SopA interacting protein (S1 Fig). In addition, this strategy identified TRIM56, a related TRIM family member (S1 Fig). The interactions between SopA and TRIM56 or TRIM65 were verified in transient co-transfection and bacterial infection experiments with epitope-tagged version of these proteins (Fig 1A and 1B). TRIM proteins are multi-domain ubiquitin E3 ligases that have been implicated in a variety of functions including innate immunity and host defense against pathogens [27–29]. Although the domain organization of the TRIM protein family varies, the general architecture is conserved, consisting of a more conserved amino-terminal tripartite motif and a variable C-terminal domain [30]. The tripartite motif, in turn, is composed of a really-interesting-new-gene (RING) domain, which carries out the E3 ubiquitin ligase function, followed by one or two B boxes and a coiled-coil domain that mediates homotypic or heterotypic interactions among TRIM family members. The C-terminal domain can contain up to 10 different modules, which most likely determines the physiological context in which TRIM family members exert their function. Phylogenetic analysis of TRIM proteins based on their tripartite motif grouped TRIM56 and TRIM65 immediately adjacent to each other [31], suggesting the presence of conserved structural features that may be relevant for their interaction with SopA. Consistent with this hypothesis, SopA did not interact with other related TRIM family members such as TRIM5, TRIM25, TRIM39 or TRIM62 (Fig 1C). Taken together these results indicate that SopA specifically interacts with TRIM65 and TRIM56.

### Interaction of SopA with TRIM-Family Proteins Requires Their RING Finger Domains

**Fig 2:**
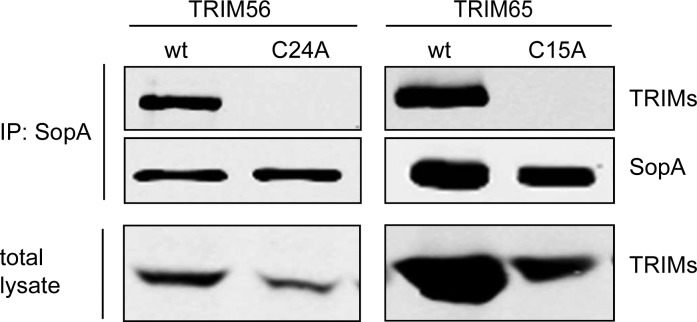
SopA interacts with the RING finger domain of TRIM65 and TRIM56. FLAG-epitope-tagged SopA^C753S^ was transiently co-expressed in HEK293T cells with M45-epitope-tagged TRIM56, TRIM65 or their RING-finger domain mutants TRIM56^C24A^ and TRIM65^C15A,^ and their interaction was analyzed by immunoprecipitation and Western immunoblotting. This experiment was repeated 3 independent times with similar results.

The RING finger domain of TRIM-family proteins binds two zinc ions through a defined motif of cysteine and histidine residues and is essential for the ubiquitination of target proteins [29]. To investigate the potential role of the RING finger domain of TRIM65 and TRIM56 in their interaction with SopA, we introduced mutations in critical cysteine residues within this domain and examined the ability of the resulting mutants to interact with SopA in transient co-transfection experiments. Introduction of a mutation in a critical cysteine residue of the RING finger domain of TRIM56 (TRIM56^C24A^) or TRIM65 (TRIM65^C15A^) completely abolished their ability to interact with SopA (Fig 2). These results indicate that SopA interaction with TRIM56 and TRIM65 requires their RING finger domain.

### SopA Enhances TRIM65 Ubiquitination

**Fig 3:**
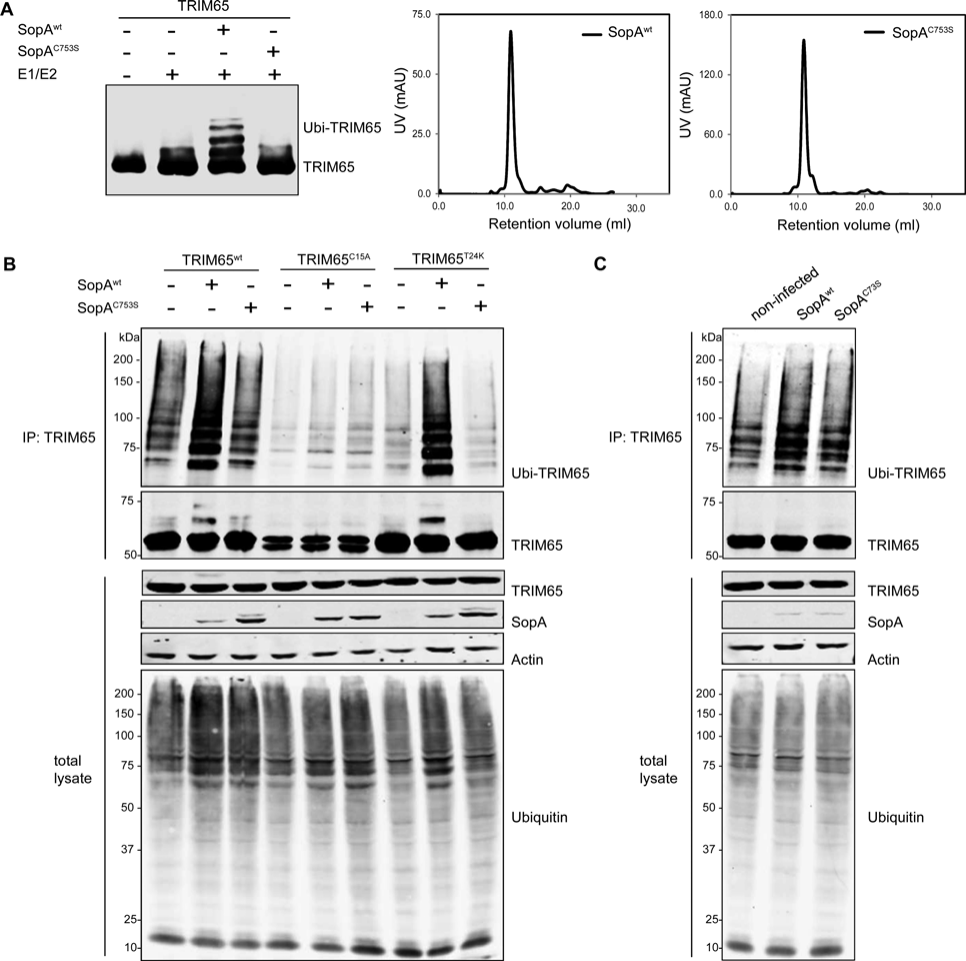
SopA enhances TRIM65 ubiquitination. (**A**) Purified FLAG-epitope-tagged TRIM65 was incubated with SopA or its catalytic mutant SopA^C753S^ in the presence of ubiquitin, ATP, E1 (UBE1) and E2 (UbcH5b) and TRIM65 ubiquitination was detected by its mobility shift in Western blot analysis with anti-FLAG antibody. The chromatographic profiles of the SopA preparations used in the assays are shown. (**B**) HEK293T cells were co-transfected with plasmids encoding HA-epitope-tagged ubiquitin, and either FLAG-epitope-tagged TRIM65, or the mutants TRIM65^C15A^ (catalytically deficient) and TRIM65^T24K^ (autoubiquitination deficient) along with plasmids encoding either SopA or its catalytic mutant SopA^C753S^. Cell lysates were evaluated for the levels of protein expression and TRIM65 ubiquitination by immunoprecipitation with anti-FLAG and Western immunoblot analysis with anti-FLAG, anti-HA, and anti-M45 antibodies, respectively. This experiment was repeated 2 independent times with similar results. (**C**) HEK293T cells were co-transfected with plasmids encoding HA-epitope-tagged ubiquitin and FLAG-epitope tagged TRIM65 and 18 hours after transfection, cells were infected with an *S*. Typhimurium strain expressing either wild type SopA or its catalytic mutant SopA^C753S^. Five hours after infection, cell lysates were evaluated for the levels of protein expression and TRIM65 ubiquitination by immunoprecipitation and Western immunoblot analysis with anti-FLAG, anti-HA, and anti-M45 antibodies, respectively. This experiment was repeated 3 independent times with similar results.

SopA has E3 ubiquitin ligase activity [25]. Therefore, we tested whether SopA could ubiquitinate its interacting proteins TRIM65 and TRIM56. Purified TRIM65 or TRIM56 were incubated with purified SopA (or the catalytic mutant SopA^C753S^) in the presence of the E1 and E2 proteins UBE1 and UbcH5b. Both, TRIM56 and TRIM65 have the capacity to autobiquitinate themselves (Fig 3A and S2A Fig). Nevertheless, we observed a significant increase in the ubiquitination of TRIM65 when incubated in the presence of wild-type SopA but not when incubated with the catalytic mutant (Fig 3A). In contrast, however, the high levels of autoubiquitinating activity of TRIM56 precluded the observation of the potential effects of the catalytic activity of SopA (S2A Fig).

We next evaluated the potential enhancement of TRIM65 and TRIM56 ubiquitination upon their co-expression with SopA and HA-epitope-tagged-ubiquitin. Consistent with the *in-vitro* results and despite the high levels of auto-ubiquitination, we observed increased TRIM65 ubiquitination after co-expression with wild type SopA but not with its catalytic mutant (Fig 3B). Furthermore, no enhancement of the ubiquitination of TRIM65^C15A^ (a mutant unable to interact with SopA, see Fig 2) was observed when co-expressed with SopA. To reduce the levels of TRIM65 auto-ubiquitination we introduced a specific mutation in its RING finger (T24K), which in other RING-type ubiquitin ligases has been shown to disrupt interaction with cognate E2 enzyme thus drastically reducing autoubiquitination [32]. We found that introduction of this mutation did not affect its ability to interact with SopA (S2C Fig). Transient co-expression of TRIM65^T24K^ with wild type SopA resulted in a marked increase in its ubiquitination (Fig 3B). In contrast, co-expression with the SopA^C753S^ catalytic mutant did not enhance TRIM65^T24K^ ubiquitination (Fig 3B). Attempts to reduce the autoubiquitination levels of TRIM56 while retaining its ability to interact with SopA were unsuccessful (S2B and S2D Fig) and therefore equivalent type of experiments could not be carried out for this TRIM protein.

We then investigated whether SopA could enhance the ubiquitination of TRIM65 during bacterial infection. Cultured cells transiently expressing TRIM65 and HA-ubiquitin were infected with *S*. Typhimurium strains expressing either wild type or the catalytic SopA^C753S^ mutant. Infected cells were evaluated for the ubiquitination levels of TRIM65 after immunoprecipitation and western blot analysis. We observed significant enhancement of TRIM65 ubiquitination after infection with *S*. Typhimurium expressing wild type SopA but not after infection with a strain expressing the catalytic mutant SopA^C753S^ (Fig 3C). Taken together, these results indicate that SopA enhances the ubiquitination of TRIM65 in a catalytically-dependent manner.

### TRIM65 Interacts with MDA5 and Enhances the Expression of Interferon-β

**Fig 4:**
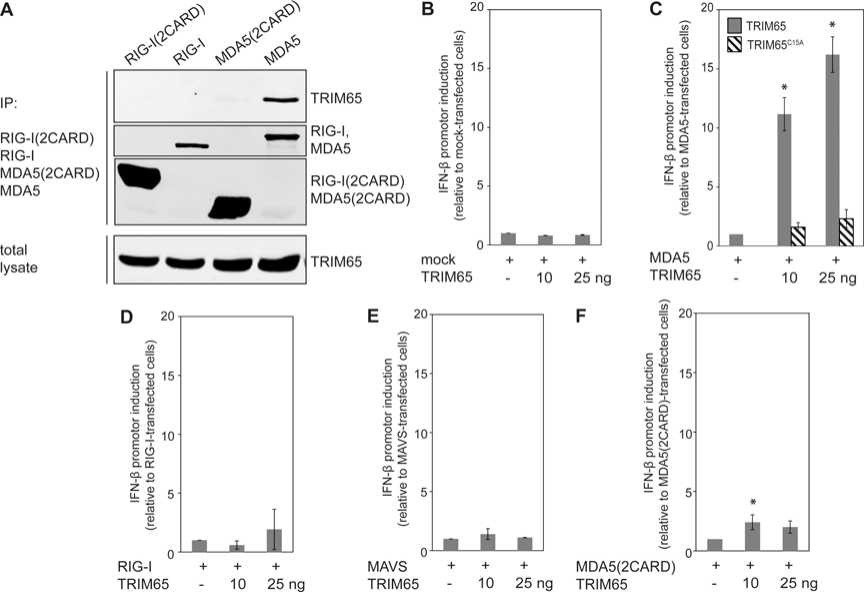
TRIM65 interacts with MDA5 and enhances MDA5-stimulated interferon-β expression. (**A**) FLAG-epitope-tagged RIG-I (2CARD) (the two CARD domains of RIG-I), RIG-I (full length), MDA5 (2CARD) (the two CARD domains of MDA5), or MDA5 (full length) were transiently expressed in HEK 293T cells together with M45-epitope-tagged TRIM65. Protein interactions were analyzed by immunoprecipitation with anti-FLAG and immunoblotting with anti-M45 and anti-FLAG antibodies. This experiment was repeated 3 independent times with similar results. (**B**-**E**) HEK293T cells were co-transfected with plasmids expressing TRIM65 or the catalytically-deficient TRIM65^C15A^ mutant along with plasmids expressing RIG-I (20 ng), MDA5 (50 ng), MDA5(2CARD) (10 ng) or MAVS (50 ng) as indicated, and the reporter plasmids expressing firefly luciferase under the control of interferon-β promoter and renilla luciferase (to standardize the transfection efficiency). The activation of the interferon-β promoter was analyzed 18 h after transfection by Dual Luciferase Reporter Assay System (Promega). Values represent the mean +/- standard deviation of the relative levels of normalized firefly luciferase from 3 independent experiments. The normalized firefly luciferase activity relative to mock-transfected cells was 10.5 ±2.9 for MDA5 only transfected cells (**C**), 11.1 ± 4.8 for RIG-I only transfected cells (**D**), 30.6 ± 17.8 for MAVS only transfected cells (**E**), and 15.4 ± 3.3 for MDA5(2CARD) only transfected cell (**F**). *: indicates statistically significant difference (p < 0.05) vs. mock transfected cells (**B**), vs. MDA5-transfected cells (**C**), vs. RIG-I-transfected cells (**D**), vs. MAVS-transfected cells (**E**) and vs. MDA5(2CARD)-transfected cells (**F**).

The role of TRIM56 in the stimulation of innate immune responses is well documented [33, 34]. In contrast, TRIM65 has been reported to be a repressor of microRNA-guided mRNA silencing and its potential involvement in innate immune responses is uncertain [35, 36]. To better understand the potential significance of its interaction with SopA, we searched for TRIM65-interacting proteins. We transduced THP-1 monocytic cells with a retroviral vector expressing FLAG epitope-tagged TRIM65 or an equally tagged irrelevant protein (as a control), and interacting proteins were identified by affinity purification combined with LC-MS/MS. To facilitate the identification of potential targets of the TRIM65 ubiquitin ligase activity, cells were treated with a proteasome inhibitor and universal Type I interferons (to stimulate the expression of interferon-induced genes) prior to affinity purification of interacting proteins. Furthermore, to identify TRIM65-interacting proteins that may be relevant during bacterial infection, cells were infected with *S*. Typhimurium prior to the analysis. We identified the melanoma differentiation-associated protein 5 (MDA5) as a TRIM65 specific interactor. This observation is intriguing since MDA5 is a member of the RNA sensor retinoic acid-inducible gene-I (RIG-I)-like receptor (RLR) family [37, 38]. This protein family is involved in the recognition of microbial nucleic acids in the cell cytoplasm with the subsequent activation of antimicrobial responses including the production of type I interferon and pro-inflammatory cytokines. We confirmed the interaction of TRIM65 with MDA5 in co-expression experiments of differentially epitope-tagged MDA5 and TRIM65 proteins (Fig 4A). In contrast, TRIM65 did not interact with the related protein RIG-I. Furthermore, TRIM65 interacted with full length MDA5 but not with a deletion consisting of just the CARD domains of MDA5. Taken together, these results indicate that TRIM65 interacts with MDA5 but not with other RLR family members such as RIG-I.

It has been previously shown that many TRIM-family proteins are able to enhance the ability of RLR family receptors to stimulate interferon-β expression [36, 39]. To investigate the potential significance of the TRIM65/MDA5 interaction, we investigated the ability of TRIM65 to modulate MDA5-stimulated interferon-β expression. As previously reported for other TRIM family members, we found that TRIM65 by itself was unable to stimulate interferon-β expression (Fig 4B) [36]. However, when co-expressed with MDA5, TRIM65 was able to enhance the ability of this RLR family member to stimulate the expression of an interferon-β reporter (Fig 4C). In contrast, TRIM65 was unable to enhance the ability of full-length RIG-I (Fig 4D) or the downstream component MAVS to stimulate the interferon-β promoter (Fig 4E). The ability of TRIM65 to stimulate MDA5-dependent interferon-β expression required its E3 ubiquitin ligase activity since a catalytic mutant (TRIM65^C15A^) was unable to stimulate interferon-β expression (Fig 4C). Co-expression of TRIM65 with the 2CARD domain of MDA5 did not result in significant stimulation of the expression of an interferon-β reporter, which is consistent with the requirement of full-length MDA5 to interact with TRIM65 (Fig 4A and 4F). These results indicate that TRIM65, through its E3 ubiquitin ligase activity, can enhance the ability of MDA5 to stimulate interferon-β expression.

### SopA Enhances TRIM65 and TRIM56-Mediated Stimulation of Interferon β Expression

**Fig 5:**
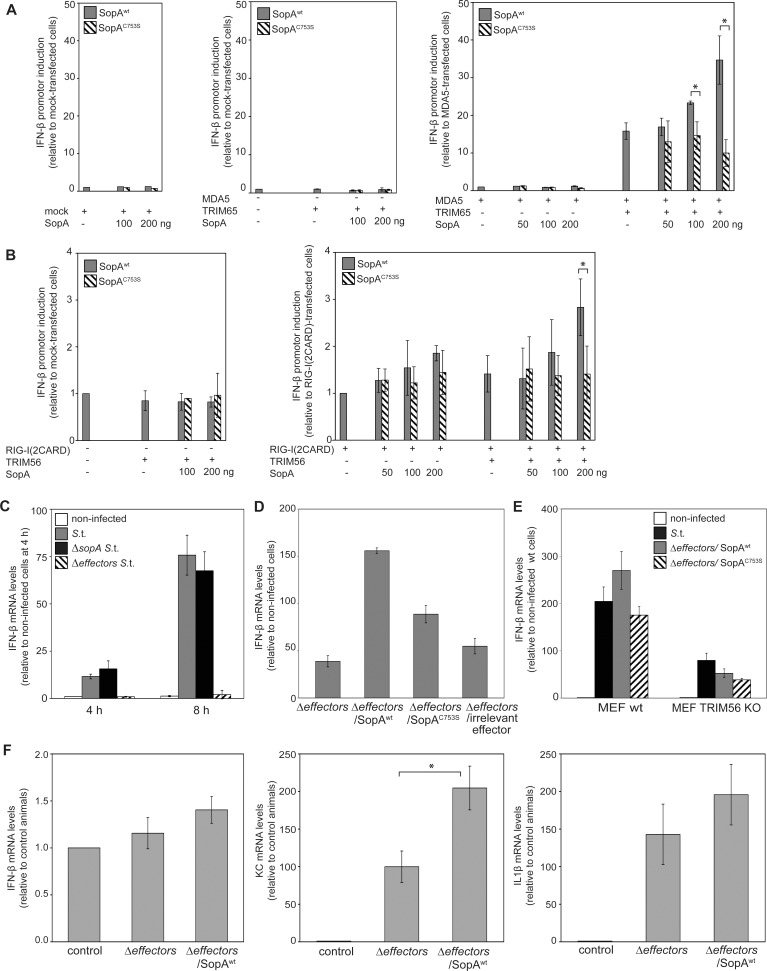
SopA enhances the ability of TRIM56 and TRIM65 to stimulate interferon-β expression. (**A**-**B**) HEK293T cells were co-transfected with plasmids expressing either wild type SopA or its catalytic mutant SopA^C753S^ along with plasmids expressing TRIM65 (25 ng), TRIM56 (10 ng), MDA5 (50ng) or RIG-I(2CARD) (4ng) as indicated, and reporter plasmids expressing firefly luciferase under the control of interferon-β promoter and renilla luciferase (to standardize the transfection efficiency). The activity of the interferon-β promoter was analyzed 18 hs after transfection with the Dual Luciferase Reporter Assay System (Promega). Values represent the mean +/- standard deviation of the relative levels of normalized firefly luciferase from 3 independent experiments. The normalized firefly luciferase activity relative to mock-transfected cells was 7.3 ± 3.9 for MDA5 only transfected cell (**B**) and 17.8 ± 9.7 for RIG-I(2CARD) only transfected cells. *: indicates statistically significant difference (*p* < 0.05) (**C**) Primary MEFs were infected with wild type *S*. Typhimurium, the *∆sopA* or the effectorless (*∆sipA ∆sptP ∆avrA ∆sopE ∆sopE2 ∆sopA ∆sopB sopD ∆sopD2 ∆slrP*) isogenic mutant strain and the levels of interferon-β gene expression were measured by qRT-PCR 4 and 8 hours after infection. Data represent the mean ± standard deviation of the *n*-fold expression of interferon-β mRNA over GAPDH relative to non-infected cells. This experiment was repeated 3 times with equivalent results. (**D**) Primary MEFs were infected with the effectorless *S*. Typhimurium strain *(∆sipA ∆sptP ∆avrA ∆sopE ∆sopE2 ∆sopA ∆sopB sopD ∆sopD2 ∆slrP*) or the effectorless strain expressing plasmid-born wild type SopA, its catalytic mutant SopA^C753S^, or an irrelevant effector. The levels of interferon-β gene expression were measured by qRT-PCR 8 hs after infection. Data represent the mean ± standard deviation of the *n*-fold expression of interferon-β mRNA over GAPDH relative to non-infected cells. The results of a representative experiment (out of 5 independent experiments) is shown. The *p* value for the difference in interferon-β mRNA levels between MEFs infected with the Δeffectors (pSopA^wt^) strain or Δeffectors (pSopA^C753S^) was *p* = 0.002 (n = 5). (**E**) Immortalized (MEF wt) and TRIM56-deficient (MEF TRIM56 KO) murine embryonic fibroblasts were infected with wild type *S*. Typhimurium or the effectorless (*∆sipA ∆sptP ∆avrA ∆sopE ∆sopE2 ∆sopA ∆sopB sopD ∆sopD2 ∆slrP*) isogenic mutant strain expressing plasmid-born wild type SopA or its catalytic mutant SopA^C753S^. The levels of interferon-β gene expression were measured by qRT-PCR 8 hours after infection. Data represent the mean ± standard deviation of the *n*-fold expression of interferon-β mRNA over GAPDH relative to non-infected cells. A representative experiment out of 7 independent experiments is shown. The *p* values for difference between the interferon-β mRNA levels in wild type MEFs infected with the Δeffectors (pSopA^wt^) or Δeffectors (pSopA^C753S^) *S* Typhimurium strains was *p* = 0.035 while the difference in TRIM56 KO cells was *p* = 0.639 (n = 7 in each category). (**F**) Effect of SopA in S. Typhimurium stimulation of pro-inflammatory cytokine expression in the mouse intestine. C57/BL6 *nramp* ^+/+^ mice were orally infected with the effectorless *S*. Typhimurium strain *(ΔsipA ΔsptP ΔavrA ΔsopE ΔsopE2 ΔsopA ΔsopB sopD ΔsopD2 ΔslrP*) (n = 6) or the effectorless strain expressing plasmid-born wild type SopA (n = 6). Seventy-two hours after infection the relative levels of the indicated cytokines in the intestine were measured by qRT-PCR. Data were normalized to the levels of GAPDH and represent the mean ± standard error relative to uninfected control animals (n = 3). *: indicates statistically significant difference (*p* < 0.05).

Ubiquitination often leads to protein degradation [40]. Therefore to investigate the consequences of SopA-mediated TRIM65 ubiquitination, we first examined whether their co-expression led to TRIM65 degradation. Transient co-expression of SopA (or its catalytic mutant) with TRIM65 did not result in a reduction of the level of TRIM65, although it led to a change in its mobility, which is consistent with its SopA-mediated ubiquitination (S3 Fig). These observations indicated that SopA-mediated ubiquitination of TRIM65 does not significantly alter its half-life and therefore this effector may not negatively affect the function of this protein. We then examined whether SopA could modulate the signaling capacity of TRIM65. We also tested the effect of SopA on TRIM56 activity since this TRIM-family member can modulate interferon-β expression and, we have shown here, also interacts with SopA. Co-expression of SopA with TRIM65 or TRIM56 did not result in increased expression of an interferon-β reporter (Fig 5A and 5B). TRIM family members by themselves are usually unable to stimulate interferon-β expression but rather, they are able to modulate interferon-β expression only in the presence of other stimulatory signals [36] (Fig 4B). Thus, it has been previously shown that TRIM56 can enhance the ability of RIG-I(2-CARD) to stimulate interferon-β expression [36] and we have shown here that TRIM65 can do the same when co-expressed with the related receptor MDA5 (Fig 4C). Therefore we co-expressed SopA (wild type or its catalytic mutant SopA^C753S^) with either TRIM65 or TRIM56 and examined the ability of these TRIM-family members to stimulate an interferon-β reporter after co-expression with either MDA5 or RIG-I(2-CARD), respectively. We found that under these conditions SopA significantly increased the ability of TRIM65 (Fig 5A) and TRIM56 (Fig 5B) to stimulate interferon-β expression. The SopA stimulatory activity was dependent on its catalytic activity since no enhancement was observed after co-expression of the SopA^C753S^ catalytic mutant (Fig 5A and 5B). These results indicate that SopA by itself is capable of stimulating the signaling capacity of TRIM65 and TRIM56.

We then examined the ability of SopA to modulate interferon-β production in the context of its delivery through the type III secretion system during *Salmonella* infection. We observed little difference between the abilities of wild type and the *∆sopA* mutant strain to stimulate interferon-β expression (Fig 5C). However, we reasoned that potential redundancies in the activities of several effector proteins might mask the agonistic potential of SopA since we observed a significant difference between wild type *S*. Typhimurium an a mutant lacking several of the SPI-T3SS effector proteins (i. e. “effectorless”) in their ability to stimulate interferon-β expression (Fig 5C). To address this issue we compared the ability of a *S*. Typhimurium effectorless strain with that of the same strain expressing SopA or the catalytic mutant SopA^C753S^. We found that cells infected with the effectorless *S*. Typhimurium strain expressing wild type SopA exhibited significantly higher stimulation capacity of interferon-β expression when compared to the effectorless strain alone or expressing the catalytic mutant SopA^C753S^ (Fig 5D). The ability of SopA to stimulate interferon-β expression was significantly impaired in the same cells rendered TRIM56-deficient using CRISPR/Cas9 technology (Fig 5E and S4 Fig). TRIM65-deficient cells, on the other hand, exhibited a complex, uninformative phenotype most likely due to its reported role in mRNA metabolism [35]. Taken together, these results indicate that *S*. Typhimurium through the catalytic activity of its effector protein SopA enhances the ability of TRIM56 and TRIM65 to stimulate interferon-β expression.

SopA has been shown to be required for the robust stimulation of inflammation in a cow model of infection [22][24], although a *S*. Typhimurium *∆sopA* mutant exhibited no observable phenotype in a mouse model of infection [24]. We nevertheless compared the *S*. typhimurium effectorless strains with the same strain expressing SopA for their potential to stimulate an inflammatory response in the mouse intestine. We found that intestine of mice infected with the strain expressing SopA had slightly increased levels of interferon-β expression although the difference did not reach statistical significance (Fig 5F). Nevertheless the expression of other pro-inflammatory cytokines was significantly increased in mice infected with the *S*. Typhimurium strain expressing SopA indicating an increased potential to stimulate an inflammatory response.

## Discussion

The bacterial enteropathogen *S*. Typhimurium has evolved redundant and specific adaptations to initiate inflammatory responses within the intestinal track that do not require the activity of highly conserved PAMPS [14–17]. The evolution of these specific adaptations has been most likely driven by at least two factors: 1) *S*. Typhimurium requires inflammation to acquire essential nutrients [12, 13]; and 2) in the intestinal epithelium most pathways that are responsible for sensing PAMPS are either absent or under tight negative regulation [6–11]. Nevertheless, once initiated by *S*. Typhimurium specific virulence factors (i. e. type III secretion effectors), the inflammatory response is most likely amplified by the activity of hard-wired canonical innate immune receptors [19–21].

Through its SPI-1 T3SS, *S*. Typhimurium delivers effector proteins that are capable of triggering in intestinal epithelial cells transcriptional responses that closely mimic those of an innate immune response that lead to the production of pro-inflammatory cytokines [14–16]. These responses are largely the result of signaling pathways triggered by the Rho-family GTPase exchange factors SopE and SopE2 and the phosphoinositide phosphatase SopB, which in a functionally redundant manner, activate Cdc42, Rac1 and RhoG [17, 41]. Signaling emanating from Rho-family GTPases result in the activation of the transcription factors AP-1, NF-κB and STAT3, which in a coordinated fashion trigger transcriptional responses leading to pro-inflammatory production [16] [18]. We report here another specific adaptation by which *S*. Typhimurium specifically modulates innate immune responses. We found that the effector protein SopA utilizes its E3 ubiquitin ligase activity to enhance interferon-β production by directly targeting the TRIM-family proteins TRIM56 and TRIM65. TRIM proteins are a large (~75) family of E3 ubiquitin ligases that have been implicated in a variety of function. Most notably, TRIM protein family members have been implicated in the regulation of various aspects of innate immunity, including NF-κB, RIG-I-like Receptors (RLRs), and Type I and Type II interferon signaling [27–29]. The distinctive feature of most members of this protein family is the presence of a tripartite motif composed of a RING domain that confers their ubiquitin ligase activity, one or two B-box motifs, and a coiled-coil region. In addition, most TRIM proteins possess a variable C-terminal domain that is thought to be responsible for the functional diversification of this protein family. Specifically TRIM56 has been implicated in the regulation of innate immune responses triggered by double stranded DNA and several viral pathogens [33, 34]. It has been proposed that TRIM56 modulates innate immune responses by ubiquinating and activating STING, a major component of the RIG-I signaling pathway [33]. In contrast, the role of TRIM65 in innate immune signaling has been so far uncertain. In fact, previous studies have linked TRIM65 to the regulation of microRNA activity by ubiquitinating the trinucleotide repeat containing protein 6 (TRNC6), which is required for RNA-mediated silencing of both micro- and short interfering-RNAs [35]. We have shown here that TRIM65 interacts with MDA5, a member of the RLR protein family, which has been implicated in the sensing of virus-derived nuclei acids [37, 38]. We also found that TRIM65 interaction with MDA5 enhances its ability to stimulate interferon-β expression. However, in contrast to TRIM56, TRIM65 was unable to stimulate RIG-I-mediated signaling, suggesting a specific role for these TRIM family members in modulating innate immune responses through different RLR receptor proteins. Thus our studies add TRIM65 to the subgroup of this large protein family that targets innate immune responses.

Expression of SopA by itself resulted in an enhancement of the ability of TRIM65 to stimulate interferon-β expression. Such enhancement required the E3 ubiquitin ligase activity of SopA since a catalytic mutant lacked any measurable stimulating ability. This is consistent with the observation that SopA was able to ubiquitinate TRIM65 both *in vitro* and *in vivo* after bacterial infection or transient co-expression. Although due to its high levels of autoubiquitination we were unable to demonstrate SopA-mediated ubiquitination of TRIM56, the observation that SopA can also enhance its ability to stimulate interferon-β expression in a catalytic-dependent manner indicate that this TRIM protein is also a relevant target of this *S*. Typhimurium effector. Consistent with this hypothesis, SopA delivered by the S. Typhimurium type III secretion system was able to stimulate interferon-β expression in cultured cells and this ability was markedly reduced in a CRISPR/Cas9-generated TRIM56-deficient cell line.

Several TRIM-family members have anti-viral functions [27]. For example, TRIM5 has been shown to restrict various retroviruses [42] and TRIM25 is essential for RIG-I-mediated antiviral activity against Influenza viruses [43]. TRIM56 itself has been implicated in the restriction of bovine viral diarrhea virus [44]. We have shown here that the bacterial pathogen *S*. Typhimurium through the activity of one T3SS effector protein has coopted the function of two TRIM-family proteins to stimulate innate immune responses. SopA has been shown to be required for the stimulation of intestinal inflammation in a cow model of infection [22–25]. Our findings provide a potential mechanistic explanation for such activity.

**Fig 6:**
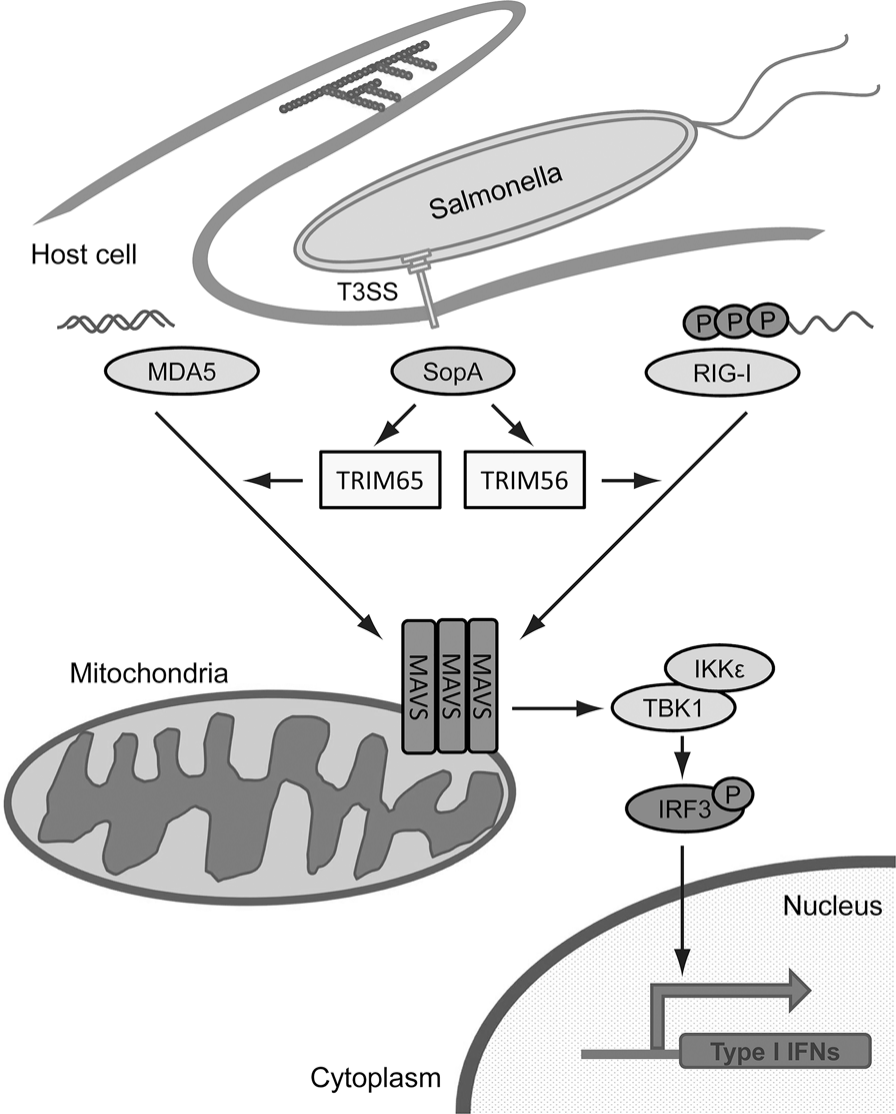
Proposed model for SopA action. After its delivery by the *S*. Typhimurium type III secretion system, SopA targets TRIM56 and/or TRIM65 and through ubiquitination, enhances their ability to modulate RIG-I and/or MDA-5-dependendant signal transduction pathways leading to pro-inflammatory cytokine production. The modulatory activity of SopA may require the prior stimulation of RIG-I and/or MDA-5 by other agonists such as nucleic acids.

RIG-I like receptors (RLRs) such as RIG-I itself and MDA5, are essential components of microbial RNA-sensing pathways [37]. TRIM56 is known to potentiate RNA-stimulated interferon-β expression through the RIG-I signaling pathway [36] and we have shown here that TRIM65 can modulate MDA5 signaling. We have also shown that SopA can enhance signaling through both TRIM56 and TRIM65, but this activity required the co-expression of the CARD domains of RIG-1 or MDA-5. This is consistent with the observation that TRIM proteins by themselves do not activate signaling pathways but they require co-expression or activation of RLRs to exert their effect [36]. It has been previously shown that during infection of non-phagocytic cells the mRNA from *S*. Typhimurium is sensed by the RIG-I pathway triggering interferon-β production [45]. Therefore it is possible that SopA may enhance these responses by positively modulating TRIM56 and TRIM65 thus leading to an increased inflammatory response (Fig 6). Consistent with this hypothesis, we found that an *S*. Typhimurium strain expressing SopA exhibited enhanced ability to stimulate the expression of pro-inflammatory cytokines in the mouse intestine. The proposed model for the function of SopA is also supported by its reported location in the mitochondria of target cells [46].

The stimulation of inflammatory responses in the intestinal track is essential for the colonization and replication of *S*. Typhimurium. We have described here a pathogen-specific adaptation to enhance the inflammatory response to *S*. Typhimurium. Understanding the mechanisms by which *Salmonella* triggers inflammation may lead to novel therapeutic and prevention strategies.

## Materials and Methods

### Cell Lines, Bacterial Strains, and Plasmids

The human epithelial cell lines Henle-407 (Roy Curtiss collection) and HeLa (ATCC), the human embryonic kidney epithelial cell line HEK 293T (ATCC) and an immortalized murine embryonic fibroblast line derived from C57BL/6 (immortalized MEFs; kind gift of P. Uchil, Yale University) were cultured in antibiotic free Dulbecco’s Modified Eagle Medium (DMEM, Gibco) supplemented with 10% bovine calf (Henle-407 and immortalized MEFs) or bovine fetal (HeLa and HEK-293T) sera. The human monocytic cell line THP-1 (ATCC) was cultured in antibiotic-free RPMI medium 1640 (Gibco) supplemented with 10% bovine fetal sera. Primary murine embryonic fibroblasts (primary MEFs; gift of P. Uchil, Yale University) were cultured in DMEM supplemented with 10% bovine fetal sera.

All plasmids and bacterial strains used in these studies are listed in S1 Table. All *S*. *enterica* serovar Typhimurium (S. Typhimurium) strains were derived from the wild type strain SL1344 [47]. A *S*. Typhimurium Δ*sopA* strain (SB1140) carrying a *sopA* deletion was constructed by standard recombinant DNA and allelic exchange procedures as previously described [48]. The effectorless strain (SB2302) carrying deletions in multiple effectors (*∆sipA*, *∆sptP*, *∆avrA*, *∆sopE*, *∆sopE2*, *∆sopA*, *∆sopB*, *sopD*, *∆sopD2*, *∆slrP*) was described previously [49]. For complementation of the *ΔsopA S*. typhimurium strains, *sopA* was placed under the control of the arabinose-inducible promoter of the expression vector pBAD24 [50] in frame with a C-terminal 3 x FLAG- or M45-epitope epitope tags. Catalytically inactive SopA^C753S^ was generated by PCR mutagenesis. For production of recombinant SopA^163-782^ and SopA^163-782, C753S,^, the *sopA* gene was subcloned into the expression vector pGEX-6P1 (Amersham) creating an amino terminal fusion to GST with a PreScission protease cleavage site. Additional 3xFLAG and 10xHis tags were engineered after the PreScission cleavage site at the N-terminus of SopA protein to yield a construct for tandem affinity purification experiments. For eukaryotic expression, wild type SopA^56-782^ and catalytic mutant SopA^56-782, C753S^ were subcloned into pRK5 vector in frame with N-terminal FLAG or M45 epitope tags. cDNAs of human TRIM56 (clone ID: 2907142, Open Biosystems), TRIM65 (clone ID: 4128330, Open Biosystems), TRIM25, TRIM5, TRIM39 and TRIM62 (kind gift of P. Uchil, Yale University) were subcloned into the eukaryotic expression vector pRK5 vector in frame with a N-terminal FLAG or M45 epitope tags. For luciferase reporter assays, TRIM56 was subcloned into SPORT6 and TRIM65 into pRK5 vectors. The mutations in the RING finger domains of TRIM56 and TRIM65 were generated by PCR mutagenesis. For retroviral transduction, TRIM65 was subcloned into pLZRS vector in frame with a N-terminal FLAG epitope. VSV-pseudotyped viruses were then produced by co-transfecting 6 μg FLAG-TRIM65 pLZRS, 6 μg pVSVG and 6 μg pGag/Pol plasmids with lipofectamine 2000 (Invitrogen) into HEK 293T cells grown in a 15-cm dish. Cell culture supernatants were collected 72 hours after transfection and used at a dilution of 1:3 to transduce THP-1 cells. Experiments with transduced cells were carried out 72 hs after transduction. Plasmids encoding human RIG-I (2CARD), RIG-I, MDA5 (2CARD), MDA5 and MAVS that were used in dual luciferase assays and co-immunoprecipitation analysis were a kind gift of E. Fikrig’s laboratory (Yale University). A pcDNA3.1-derived plasmid expressing Cas9 and a pUC19-derived plasmid expressing gRNA (used for the cloning of TRIM56 and TRIM65 target sequences) were a kind gift Dr. Zheng Fan Jiang (Peking University).

### Generation of TRIM56 and TRIM65 Deficient Murine Embryonic Fibroblast

TRIM56 and TRIM65 deficient murine embryonic fibroblast (MEF TRIM56 KO and MEF TRIM65 KO) were generated using CRISPR/Cas9 mediated homologous repair [51, 52]. Immortalized MEFs were seeded on a 6-well plate (3 x 10^5^ cells per well) and transfected using Lipofectamine 2000 (Invitrogen) with a gRNA expression vector (1 μg), Cas9-expressing plasmid (0.5 μg), a plasmid carrying a puromycin resistance gene (1 μg), and a homology-repair template in which the target sequences of TRIM56 and TRIM65 were replaced by STOP codons within each of the 3 reading frames yielding the addition of an extra 60 bp to the sequence (2.5 μg). The target sequence for the TRIM56 gene was CTGGCACAACTGGACATCGG, and the primer sequences to generate the flanking regions with STOP codons were: forward, TGGACAAAGACTGCGGGACC, reverse, ACAGTCCTGGCAATAGGTATGTAGGC, and forward, TCAGGTCCGCTGCCCCG, reverse, TTCAGCTGCCTCCTCCACCT, respectively. The target sequence for the TRIM65 gene was CCATCTGCTTAGGTCGCTAC, and the primer sequences to generate the flanking regions with STOP codons were: forward, GGTGAGCAGAAGGTGCCCAG, reverse, AGCAAGTCACCACGTCCTCCTC, and forward, GACCCGGTGACGCTGCC, reverse, GCCCGGTCTCCCGAAGAAAC. Forty-eight hours after transfection cells were plated onto 10 cm dishes and grown in the presence of puromycin (0.5 μg / ml) for 4 days. After selection, 5 × 10^2^–1 × 10^3^ puromycin-resistant MEFs were seeded onto a 15 cm dish and cultured until visible colonies were observed. Colonies were subsequently transferred onto 24-well plates using sterile filter disks soaked in trypsin and after further multiplication, cells were analyzed by PCR for disruption of the TRIM56 and TRIM65 genes. Primers for the verification of TRIM56 gene disruption were: forward, AGCAGCGATTTCCTAGCCTG and reverse, GCACAATCTCCCGACACTCG. These primers yield a 148 bp PCR product in wild type cells and a 208 bp product in TRIM56-disrupted cells (S4 Fig) Primers for the verification of TRIM65 gene disruption were: forward, GGAGGAGGACGTGGTGACTT and reverse, TTCGCAGGAACGCCATGAAT. These primers yield a 118 bp PCR product in wild type cells and a 178 bp product in TRIM65-disrupted cells (S4 Fig).

### Bacterial Infections

Overnight cultures of the different *S*. Typhimurium strains were diluted 1:20 in LB broth containing 0.3M NaCl, and grown until they reached an OD600 of 0.9. Unless specified, cell lines at a confluency of 80% were infected with the *S*. Typhimurium strains at an MOI of 30 for 1 h in Hank’s buffered salt solution (HBSS), and subsequently incubated in DMEM supplemented with gentamicin (100 μg/ml) to kill extracellular bacteria. After 30 min, the concentration of gentamicin was decreased to 10 μg/ml. When required, 0.2% L-arabinose was present throughout the experiment to induce expression of SopA under the control of the arabinose-inducible p*araABC* promoter.

### Protein Expression and Purification

All type III secretion effector proteins were expressed in *E*. *coli* DH5α from pGEX-6P1 (Amersham) expression vectors after addition of the inducer IPTG (0.1 mM) and growth at 25°C. Bacterial cells were lysed by French Press, and effectors were purified from soluble fractions with Glutathione-Sepharose 4B (Amersham) and eluted with 10 mM reduced glutathione. Purified SopA protein preparations were dialysed overnight into 20 mM Hepes (pH 7.4), 150 mM NaCl, and when required, SopA was released by cleavage with PreScission protease. All preparations were subjected to centrifugation (100,000 g for 1 h) to remove any insoluble materials and further purified using a Superdex 200 10/300 GL column on an AKTA UPC10 purification system. TRIM56 and TRIM65 proteins with N-terminal FLAG epitope tags were purified using anti-FLAG M2 agarose (Sigma) after transient transfection of HEK 293T cells seeded in 10 cm dishes with 4 μg of FLAG-TRIM56 and FLAG-TRIM65 pRK5 expression vectors. TRIM proteins were eluted with FLAG peptide (150 ng/μl) in 50 mM Tris-HCl (pH 7.5), 50 mM NaCl and subsequently used for *in vitro* ubiquitination assays.

### Affinity Purification and Mass Spectrometry Analysis

For tandem affinity purification of SopA-interacting proteins, 1.5 × 10^8^ HEK 293T cells or 7.5 × 10^9^ HeLa cells were lysed for 10 min in 4 ml of ice cold buffer containing 20 mM Hepes (pH 7.4), 150 mM NaCl, 0.3% Triton-X-100 and Complete Mini protease inhibitors (EDTA free, Roche). The nuclei and insoluble cellular components were removed by centrifugation at 5,000 × g for 5 min, and supernatants were further clarified using Glutathione-Sepharose 4B (Amersham). Fifty μg of SopA^163-782, C753S^ or unrelated type III effector proteins (to control nonspecific binding) were then added to the supernatant and binding of SopA-interacting proteins from cell lysates to SopA was allowed for 3 h. Effectors were then purified using Glutathione-Sepharose 4B (Amersham) and anti-FLAG M2 agarose (50% slurry, Sigma) with washing steps employing ice cold buffer containing 20 mM Hepes (pH 7.4), 150 mM NaCl and 0.1% Triton-X-100. In the final step, the effector proteins were eluted using 3x FLAG peptide (150 ng/μl).

For immunoaffinity purification of SopA-interacting proteins during bacterial infection, 7.5 × 10^9^ Henle 407 cells were infected for 5h at an MOI 1:30 with *S*. Typhimurium expressing arabinose-inducible FLAG-epitope-tagged SopA^C753S^ or an equally tagged control effector protein. For immunoaffinity purification of TRIM65-interacting proteins, 7.5 × 10^9^ THP1 cells transduced with FLAG-TRIM65 or an irrelevant FLAG-tagged protein were treated overnight with 10 ng/ml PMA, 0.1 μM MG-132, and 50 EU/ml Universal type I Interferon (PBL interferon) and infected with *S*. Typhimurium at an MOI 1:25 for 1 h. Infected cells were then lysed in 4 ml of ice cold buffer containing 20 mM Hepes (pH 7.4), 150 mM NaCl, 0.2% Triton-X-100 and Complete Mini protease inhibitors (EDTA free, Roche) and samples were clarified by centrifugation at 5,000 × g for 5 minute. The supernatants were used for immunoprecipitation using prewashed anti-FLAG M2 agarose (50% slurry, Sigma). After O/N incubation under rotation at 4C, immune complexes were collected by centrifugation at 1,500 × g for 3 min, washed three times with buffer containing 20 mM Hepes (pH 7.4), 150 mM NaCl and 0.1% Triton-X-100 and eluted with 3x FLAG peptide (150 ng/μl). The eluates were run on SDS-PAGE, and each lane was separated in three gel slices that were excised and subjected to in-gel trypsin digestion O/N as described previously [53]. Peptides that were extracted from the gel matrix were resuspended in aqueous buffer and subjected to LC-MS/MS analysis as previously described [53].

### Co-immunoprecipitation Analysis

For analysis of protein interactions, 7.5 × 10^6^ HEK 293T cells seeded on 10 cm dishes were transiently transfected using Lipofectamine 2000 (Invitrogen) with 2 μg of plasmid encoding indicated FLAG-epitope-tagged protein together with 2 μg of plasmid encoding indicated M45-epitope-tagged protein, or solely with 4 μg of pRK5 vector encoding M45-epitope-tagged TRIM56 or TRIM65. Twenty-four hours post-transfection, the cells were either infected with *S*. Typhimurium expressing arabinose-inducible FLAG-tagged SopA^C753S^ which was followed by cell lysis or lysed directly in 1 ml of ice cold buffer containing 20 mM Hepes (pH 7.4), 150 mM NaCl, 0.2% Triton-X-100 and Complete Mini protease inhibitors (EDTA free, Roche). The lysate was clarified by centrifugation at 5,000 × g for 5 min, and further used for immunoprecipitation using 20 μl of prewashed anti-FLAG M2 agarose (50% slurry, Sigma) with 2 h rotation. Immune complexes were collected by centrifugation at 1500 × g for 3 min, washed three times with 1 ml of cold lysis buffer and eluted by 3x FLAG peptide (150 ng/μl). Samples were analyzed by SDS-PAGE (10% gel) and western blot with antibodies against M45 and FLAG.

### Ubiqutination of TRIM56 and TRIM65

*In vitro* ubiquitination experiments were carried out in a reaction mixture (40 μl) containing 50 mM Tris-HCl (pH 7.5), 50 mM NaCl, 10 mM MgCl_2_, 5 mM ATP, 0.1 mM DTT, 100 μM ubiquitin (Boston Biochem), 50 nM UBE1 (Boston Biochem), 200 nM UbcH5b (Boston Biochem) and purified FLAG-TRIM56 or FLAG-TRIM65. The reactions were supplemented with 200 nM of recombinant SopA^163-782^ or SopA^163-782 C753S^ as indicated. Following 90 min incubation at 37°C, the reactions were quenched with SDS sample buffer containing 100 mM DTT and analyzed by SDS-PAGE (10% gel) and western blot with antibodies against FLAG.

For analysis of TRIM ubiquitination upon SopA co-expression, HEK 293T seeded at 2 × 10^6^ per well on a 6-well plate were transiently transfected with plasmids encoding indicated FLAG-epitope-tagged TRIM protein (0.5 μg), HA-ubiquitin (1 μg), and M45-epitope-tagged SopA^56-782^ (wild type or catalytic mutant C753S, 1 μg). For analysis of TRIM ubiquitination upon SopA delivery through T3SS, HEK 293T cells were transfected with plasmids encoding indicated FLAG-epitope-tagged TRIM protein (0.5 μg) and HA-ubiquitin (1 μg) which was followed by 5 h infection with *S*. Typhimurium expressing arabinose-inducible M45-tagged SopA or SopA^C753S^. Twenty four hours post-transfection, the cells were lysed in 1 ml of ice cold buffer containing 20 mM Hepes (pH 7.4), 150 mM NaCl, 0.2% Triton-X-100, 20 mM NEM, 1 μM MG-132 and Complete Mini protease inhibitors. The lysates were clarified by centrifugation at 5,000 × g for 5 min, and used for immunoprecipitation using anti-FLAG M2 agarose. After 2 hs incubation at 4°C, immune complexes were collected by centrifugation at 1500 × g for 3 min, washed four times with 1 ml of cold lysis buffer, and resuspended in SDS sample buffer. Samples were analyzed by SDS-PAGE (10% gel) and western blot with antibodies against HA and FLAG.

### Dual-Luciferase Assay

HEK 293T seeded at 2 × 10^5^ in a 24-well plate were transfected using Lipofectamine 2000 (Invitrogen) with indicated amounts of plasmids expressing TRIM proteins, or expressing the inducers of immune signaling pathways (RIG-I, MDA5, MAVS, or RIG-I (2CARD), and the SopA effector along with the reporter plasmid and an internal transfection control. The reporter plasmid encodes the firefly luciferase under the control of interferon-β promoter and the internal transfection control reporter plasmid (pRL-TK, Promega) expresses *Renilla* luciferase. The total amount of DNA was equalized to 500 ng for each transfection using pRK5 vector. Cells were lysed 24 h post transfection with passive lysis buffer (Promega), and the luciferase activity was measured using a Dual-Glo luciferase assay system (Promega) in a Berthold multiwell luminometer. Firefly luciferase data were normalized to *Renilla* luciferase readings in each well, and the data were represented as the fold change compared to control for at least three experiments carried out on separate days.

### Statistical Analysis

Statistical significance was calculated by a one-tail distributed paired Student’s t-test. Resulting *p*-values of less than 0.05 were considered significantly different.

### Quantitative Real-Time PCR

MEFs seeded at 4 × 10^5^ on a 6-well plate were infected with the indicated *S*. typhimurium strains at MOI 1:20. Four or 8 hours post-infection, total RNA was isolated from cells using RNeasy Mini kit (Qiagen), digested with DNAse I (Invitrogen) and used as a template for cDNA synthesis using the iScript reverse transcriptase (Bio-Rad). Normalized interferon-β transcript levels were determined using iQ SYBR Green Supermix (Bio-Rad), in an iCycler real time PCR machine (Bio-Rad) with specific murine interferon-β and GAPDH mRNA primer sets (interferon-β forward 5′-CAGCTCCAAGAAAGGACGAAC-3′ and reverse 5′-GGCAGTGTAACTCTTCTGCAT-3′, GAPDH forward 5′-AGGTCGGTGTGAACGGATTTG-3′ and reverse 5′-GGGGTCGTTGATGGCAACA-3′), that have been designed by PrimerBank (http://pga.mgh.harvard.edu/primerbank/).

### Mouse Infections and Quantitative PCR of Animal Tissues

Mouse infections, tissue extraction and quantitative PCR analysis was carried out as previously described [54]. Briefly, groups of age- and sex-matched 8–12 week-old C57BL/6 mice carrying a wild type allele of *Nramp1* (*Slc11a1*) were administered 100 μl of a 100 μg/ml solution of streptomycin 24 hs prior to bacterial infection. Food was removed 4 hs prior to inoculation, and mice were administered (by stomach gavage) 100 μl of 10% bicarbonate solution (to buffer the stomach pH) followed by the administration of 10^8^ c. f. u. of the indicated bacterial strains in 100 μl PBS. Three days after infection, total RNA from mouse tissues (cecum) were isolated using TRIzol (Invitrogen) reagent according to the manufacture’s protocol and were reversed-transcribed with the iScript reverse transcriptase (BIORAD). Quantitative PCR was performed using iQ SYBR Green Supermix (BIORAD) in an iCycler real time PCR machine (Bio-Rad). All animal experiments were conducted according to protocols approved by Yale University’s Institutional Animal Care and Use Committee.

### Ethics Statement

All animal experiments were conducted according to protocols approved by Yale University’s Institutional Animal Care and Use Committee under protocol number 2013–07858. The IACUC is governed by applicable Federal and State regulations, including those of the Animal Welfare Act (AWA), Public Health Service (PHS), and the United States Department of Agriculture (USDA) and is guided by the U.S. Government Principles for the Utilization and Care of Vertebrate Animals Used in Testing, Research and Training.

## Supporting Information

S1 Fig Identification of SopA-interacting proteins by LC-MS/MS. **(A)** Chromatographic profiles of the purified effectors used for affinity-purification of interacting proteins. Purified effector proteins were characterized using Superdex 200 10/300 GL column on an AKTA purification system. Coomassie blue stained SDS-PAGE of the purified effector used in the affinity purification studies are shown as insets. **(B)** Peptides corresponding to the SopA-interacting proteins identified by LC-MS/MS are shown. Peptides identified by affinity purification using purified proteins are depicted in red, peptides identified in bacterial infection experiments are depicted in green, and peptides identified in both type of experiments are depicted in orange.
(TIF)

S2 Fig Auto-ubiquitination activity of TRIM56 and interaction of SopA and RING finger domain mutants of TRIM65 and TRIM56. (**A**) Purified TRIM56 was incubated with SopA or its catalytic mutant SopA^C753S^ in the presence of ubiquitin, ATP, E1 (UBE1) and E2 (UbcH5b) and TRIM56 ubiquitination was detected by its mobility shift in Western blot analysis. (**B**) HEK 293T cells were co-transfected with plasmids encoding HA-ubiquitin, FLAG-TRIM56, or FLAG-TRIM56^C24A^ (catalytically deficient mutant) along with plasmids encoding either SopA or its catalytic mutant SopA^C753S^. Cell lysates were evaluated for the levels of TRIM56 ubiquitination by immunoprecipitation and immunoblot analysis with anti-FLAG and anti-HA antibodies, respectively. (**C**) FLAG-epitope-tagged SopA^C753S^ was transiently co-expressed in HEK293T cells with M45-epitope-tagged TRIM65 or RING-finger domain mutants TRIM65^C15A^ and TRIM65^T24K^, and SopA-TRIM65 interactions were analyzed by immunoprecipitation and Western immunoblotting. (**D**) FLAG-epitope-tagged SopA^C753S^ was transiently co-expressed in HEK293T cells with M45-epitope-tagged TRIM56 or RING-finger domain mutants TRIM56^C24A^, TRIM56^T33K^, TRIM56^I23E^ and TRIM56^L48E^, and SopA-TRIM56 interactions were analyzed by immunoprecipitation and Western immunoblotting.
(TIF)

S3 Fig Co-expression of TRIM65 with SopA does not lead to TRIM65 degradation. Plasmid for expression of FLAG-epitope-tagged TRIM65 (100 ng) was co-transfected into 2 × 10^5^ HEK293T cells with increasing amounts of plasmids for expression of M45-epitope-tagged SopA or its catalytic mutant SopA^C753S^ (100, 200, 300 or 400 ng). Twenty-four h after transfection, whole cell lysates were immunoblotted with anti-FLAG, and anti-M45.
(TIF)

S4 Fig Verification of the TRIM56 and TRIM65 deficient murine embryonic fibroblast by PCR analysis. Shown are the profiles of amplified DNA fragments from the indicated cells. Expected PCR products for TRIM56 gene are 148 bp in wild type and 208 bp in TRIM56-disrupted cells, and expected PCR products for TRIM65 gene are 118 bp in wild type and 178 bp in TRIM65-disrupted cells.
(TIF)

S1 Table Strains and plasmids used in this study. (PDF)
