# Supplementary figures and images for: The Salmonella Effector Protein SopA Modulates Innate Immune Responses by Targeting TRIM E3 Ligase Family Members
Source: PLoS Pathog. 2016 Apr 8;12(4):e1005552. doi: 10.1371/journal.ppat.1005552 (PMC4825927; doi:10.1371/journal.ppat.1005552)

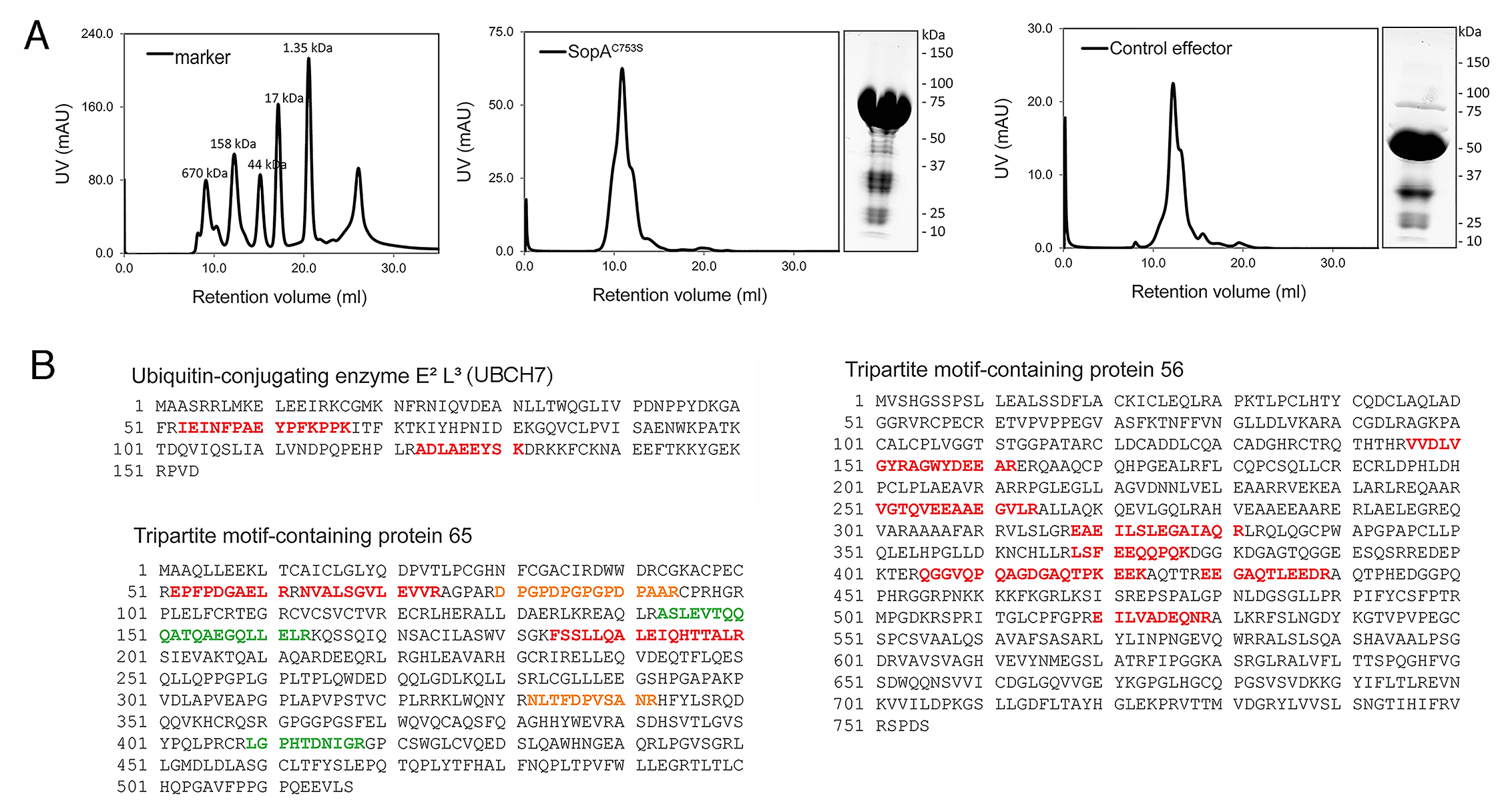

Supplement: S1 Fig — (A) Chromatographic profiles of the purified effectors used for affinity-purification of interacting proteins. Purified effector proteins were characterized using Superdex 200 10/300 GL column on an AKTA purification system. Coomassie blue stained SDS-PAGE of the purified effector used in the affinity purification studies are shown as insets. (B) Peptides corresponding to the SopA-interacting proteins identified by LC-MS/MS are shown. Peptides identified by affinity purification using purified proteins are depicted in red, peptides identified in bacterial infection experiments are depicted in green, and peptides identified in both type of experiments are depicted in orange. (TIF) [file ppat.1005552.s001.tif]

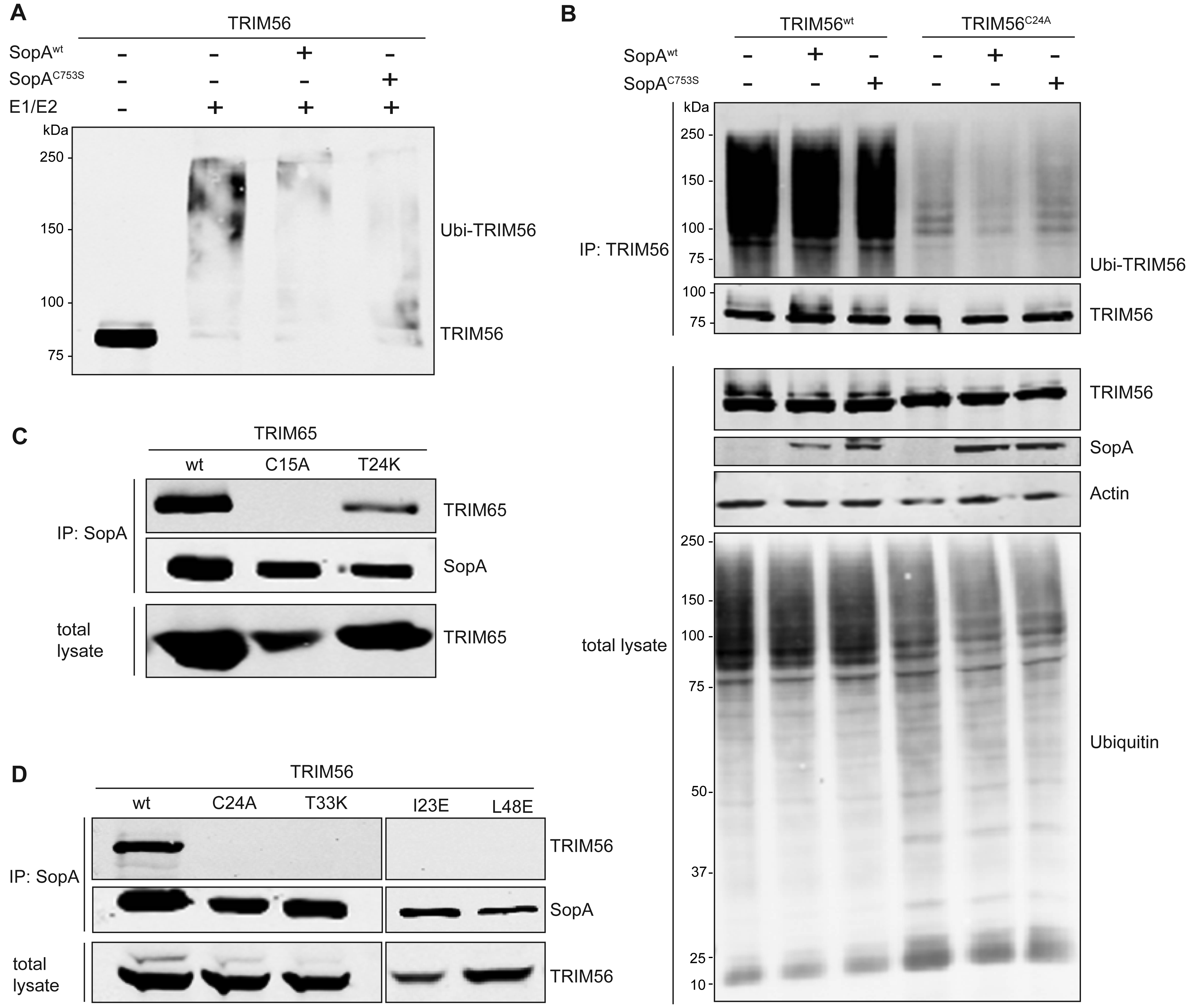

Supplement: S2 Fig — (A) Purified TRIM56 was incubated with SopA or its catalytic mutant SopAC753S in the presence of ubiquitin, ATP, E1 (UBE1) and E2 (UbcH5b) and TRIM56 ubiquitination was detected by its mobility shift in Western blot analysis. (B) HEK 293T cells were co-transfected with plasmids encoding HA-ubiquitin, FLAG-TRIM56, or FLAG-TRIM56C24A (catalytically deficient mutant) along with plasmids encoding either SopA or its catalytic mutant SopAC753S. Cell lysates were evaluated for the levels of TRIM56 ubiquitination by immunoprecipitation and immunoblot analysis with anti-FLAG and anti-HA antibodies, respectively. (C) FLAG-epitope-tagged SopAC753S was transiently co-expressed in HEK293T cells with M45-epitope-tagged TRIM65 or RING-finger domain mutants TRIM65C15A and TRIM65T24K, and SopA-TRIM65 interactions were analyzed by immunoprecipitation and Western immunoblotting. (D) FLAG-epitope-tagged SopAC753S was transiently co-expressed in HEK293T cells with M45-epitope-tagged TRIM56 or RING-finger domain mutants TRIM56C24A, TRIM56T33K, TRIM56I23E and TRIM56L48E, and SopA-TRIM56 interactions were analyzed by immunoprecipitation and Western immunoblotting. (TIF) [file ppat.1005552.s002.tif]

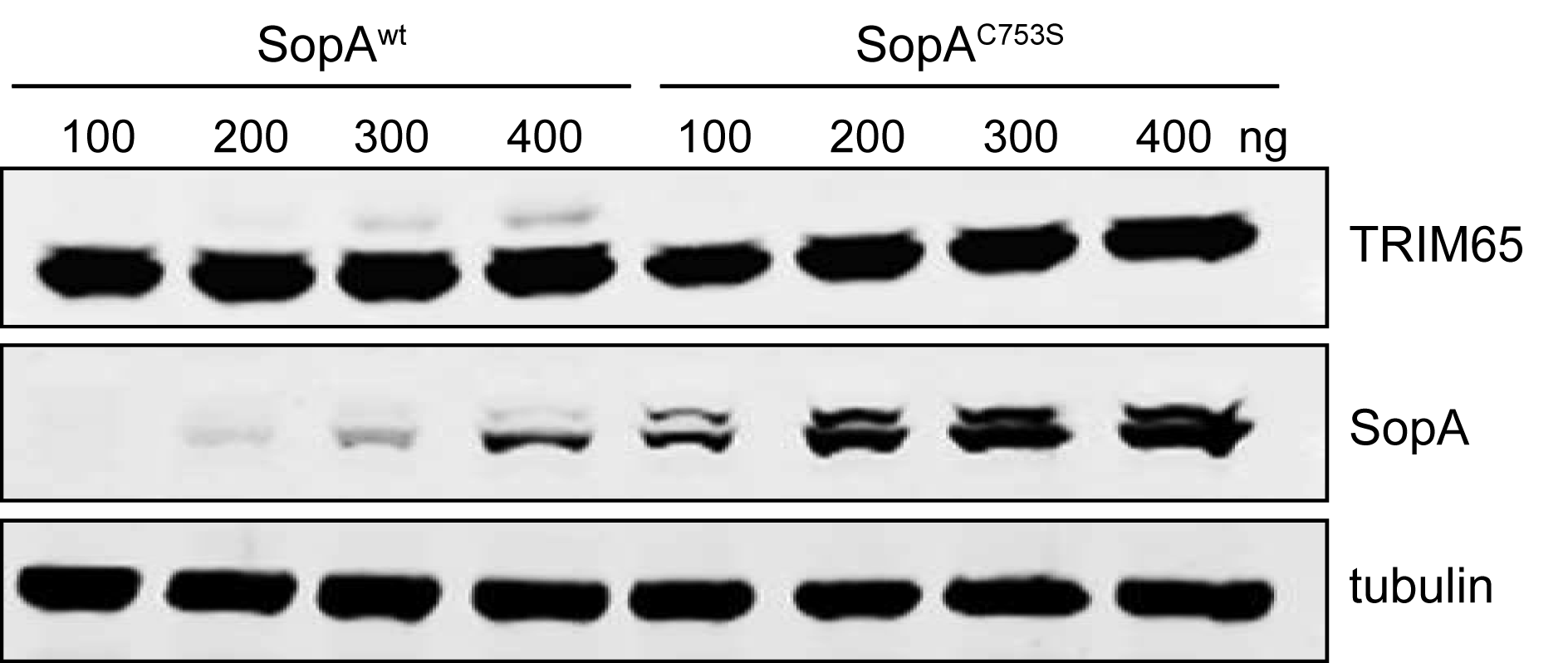

Supplement: S3 Fig — Plasmid for expression of FLAG-epitope-tagged TRIM65 (100 ng) was co-transfected into 2 × 10^5^ HEK293T cells with increasing amounts of plasmids for expression of M45-epitope-tagged SopA or its catalytic mutant SopAC753S (100, 200, 300 or 400 ng). Twenty-four h after transfection, whole cell lysates were immunoblotted with anti-FLAG, and anti-M45. (TIF) [file ppat.1005552.s003.tif]

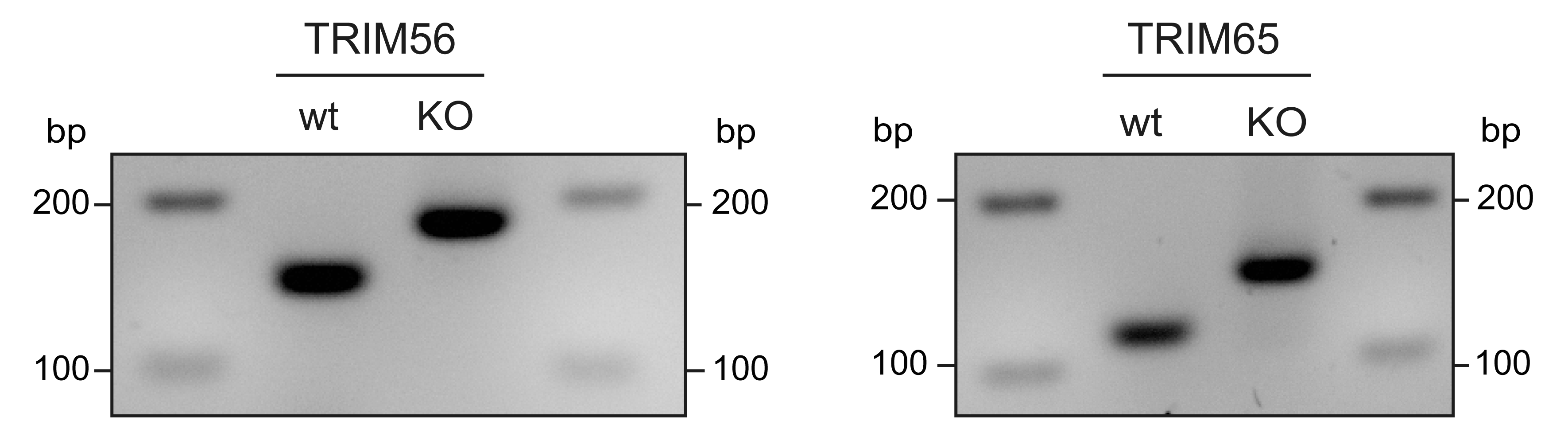

Supplement: S4 Fig — Shown are the profiles of amplified DNA fragments from the indicated cells. Expected PCR products for TRIM56 gene are 148 bp in wild type and 208 bp in TRIM56-disrupted cells, and expected PCR products for TRIM65 gene are 118 bp in wild type and 178 bp in TRIM65-disrupted cells. (TIF) [file ppat.1005552.s004.tif]
