# Supplementary material for: The Salmonella Effector Protein SopA Modulates Innate Immune Responses by Targeting TRIM E3 Ligase Family Members
Source: PLoS Pathog. 2016 Apr 8;12(4):e1005552. doi: 10.1371/journal.ppat.1005552 (PMC4825927; doi:10.1371/journal.ppat.1005552)
Supplement: S1 Table — (PDF) [file ppat.1005552.s005.pdf]

Table S1: Plasmids used in this study

| Encoded protein | Vector | Tag | SB number |
|---|---|---|---|
| SopA | pBAD24 | 3'- 3xFLAG | pSB5201 |
| SopA$^{C753S}$ | pBAD24 | 3'- 3xFLAG | pSB5202 |
| SopA | pBAD24 | 3'- M45 | pSB5204 |
| SopA$^{C753S}$ | pBAD24 | 3'- M45 | pSB5205 |
| SopA | pBAD24 | without tag | pSB5206 |
| SopA$^{C753S}$ | pBAD24 | without tag | pSB5207 |
| SopA$^{163\text{-}782,\ C753S}$ | pGEX-6PI | 5'- GST-3xFLAG-10xHis | pSB5208 |
| SopA$^{163\text{-}782}$ | pGEX-6PI | 5'- GST | pSB5211 |
| SopA$^{163\text{-}782,\ C753S}$ | pGEX-6PI | 5'- GST | pSB5212 |
| SopA$^{56\text{-}782}$ | pRK5 | 5'- FLAG | pSB5213 |
| SopA$^{56\text{-}782,\ C753S}$ | pRK5 | 5'- FLAG | pSB5214 |
| SopA$^{56\text{-}782}$ | pRK5 | 5'- M45 | pSB5215 |
| SopA$^{56\text{-}782,\ C753S}$ | pRK5 | 5'- M45 | pSB5216 |
| SopA | pWSK | Without tag | pSB5241 |
| TRIM56 | pRK5 | 5'- FLAG | pSB5217 |
| TRIM56$^{C24A}$ | pRK5 | 5'- FLAG | pSB5218 |
| TRIM56 | pRK5 | 5'- M45 | pSB5219 |
| TRIM56$^{C24A}$ | pRK5 | 5'- M45 | pSB5220 |
| TRIM56$^{T33K}$ | pRK5 | 5'- M45 | pSB5221 |
| TRIM56$^{I23E}$ | pRK5 | 5'- M45 | pSB5222 |
| TRIM56$^{L48E}$ | pRK5 | 5'- M45 | pSB5223 |
| TRIM56 | pSPORT6 | without tag | pSB5224 |
| TRIM65 | pRK5 | 5'- FLAG | pSB5225 |
| TRIM65$^{C15A}$ | pRK5 | 5'- FLAG | pSB5226 |
| TRIM65$^{T24K}$ | pRK5 | 5'- FLAG | pSB5227 |
| TRIM65 | pRK5 | 5'- M45 | pSB5228 |
| TRIM65$^{C15A}$ | pRK5 | 5'- M45 | pSB5229 |
| TRIM65$^{T24K}$ | pRK5 | 5'- M45 | pSB5230 |
| TRIM65 | pRK5 | without tag | pSB5231 |
| TRIM65$^{C15A}$ | pRK5 | without tag | pSB5232 |
| TRIM65 | pLZRS | 5'- FLAG | pSB5233 |
| TRIM25 | pRK5 | 5'- M45 | pSB5235 |
| TRIM5 | pRK5 | 5'- M45 | pSB5236 |
| TRIM39 | pRK5 | 5'- M45 | pSB5237 |
| TRIM62 | pRK5 | 5'- M45 | pSB5238 |
